# How Can We Manage Gallbladder Lesions by Transabdominal Ultrasound?

**DOI:** 10.3390/diagnostics11050784

**Published:** 2021-04-26

**Authors:** Shinji Okaniwa

**Affiliations:** Department of Gastroenterology, Iida Municipal Hospital 1, Iida, Nagano 395-8502, Japan; okaniwa@cocoa.ocn.ne.jp; Tel.: +81-265-21-1255; Fax: +81-265-21-1266

**Keywords:** gallbladder carcinoma, ultrasound (US), differential diagnosis, polypoid lesion, wall thickening, high-resolution US (HRUS), color Doppler imaging, contrast-enhanced US (CEUS), high-frequency transducer

## Abstract

The most important role of ultrasound (US) in the management of gallbladder (GB) lesions is to detect lesions earlier and differentiate them from GB carcinoma (GBC). To avoid overlooking lesions, postural changes and high-frequency transducers with magnified images should be employed. GB lesions are divided into polypoid lesions (GPLs) and wall thickening (GWT). For GPLs, classification into pedunculated and sessile types should be done first. This classification is useful not only for the differential diagnosis but also for the depth diagnosis, as pedunculated carcinomas are confined to the mucosa. Both rapid GB wall blood flow (GWBF) and the irregularity of color signal patterns on Doppler imaging, and heterogeneous enhancement in the venous phase on contrast-enhanced ultrasound (CEUS) suggest GBC. Since GWT occurs in various conditions, subdividing into diffuse and focal forms is important. Unlike diffuse GWT, focal GWT is specific for GB and has a higher incidence of GBC. The discontinuity and irregularity of the innermost hyperechoic layer and irregular or disrupted GB wall layer structure suggest GBC. Rapid GWBF is also useful for the diagnosis of wall-thickened type GBC and pancreaticobiliary maljunction. Detailed B-mode evaluation using high-frequency transducers, combined with Doppler imaging and CEUS, enables a more accurate diagnosis.

## 1. Introduction

As ultrasound (US) is a simple and noninvasive procedure without radiation exposure, it is widely used for cancer screening and health checkups [1,2]. Both gallbladder (GB) polypoid lesions (GPLs) and wall thickening (GWT) are common US findings, and GB carcinomas (GBCs) are also detected incidentally in patients with no symptoms. According to the systematic review, malignant GPLs had an incidence of just 0.57% [3].

The management of GB lesions can be divided into detection, differentiation, and evaluation of extension. GBC is often diagnosed at an advanced stage because of being asymptomatic in early stages, its aggressive nature, and its ability to spread rapidly. However, the detection and resection rate for GBC in the mass screening of 204,099 persons is 0.040% (81 cases) and 88%, respectively [2], and US can depict 55% or more of GBC as tumors [4]. Thus, US is recommended as the first step modality for patients with suspicion of GB abnormalities [5,6], including cholelithiasis, inflammation, and malignancy.

However, the sensitivity and accuracy of US are highly dependent on the diagnostic skill of sonographers and conditions of patients. In addition, both detection and differentiation of lesions become more challenging in complicated conditions of GB, such as acute and chronic inflammation and inclusion of stones or debris.

This review classifies various GB lesions according to their US appearance and reviews the current status of differential diagnosis by US findings.

## 2. Tips for GB Evaluation

**Figure 1:**
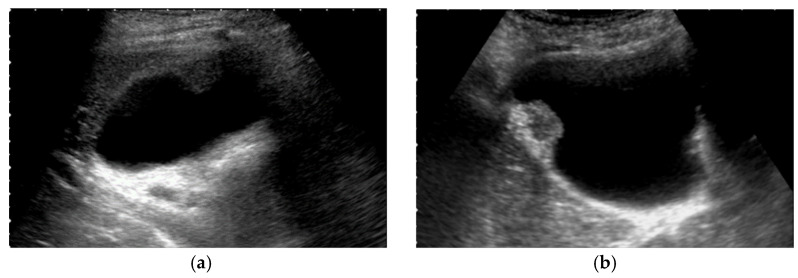
Alteration of US images with postural changes. In the supine position, GBC in the fundus was obscured by reverberation artifacts (**a**), but a high-frequency probe can delineate the GBC in the left lateral decubitus position (**b**).

The GB is an elliptical or pear-shaped sac-like organ attached to the inferior surface of the liver and easily changes its shape with food intake and postural position, which makes it difficult to visualize the entire GB. To avoid overlooking lesions, especially in the neck and fundus, postural changes such as left lateral decubitus or sitting position should add to ordinal supine imaging (Figure 1). Furthermore, applying the probe diagonally to the abdominal wall can manage reverberations that obscure lesions in the fundus [7]. It is also effective to adopt a shallow depth (about 6–8 cm) or employ a zoom magnification sufficient to recognize the whole GB, to pick up small lesions that are difficult to detect with a normal depth of field (12–14 cm).

Compared with low-frequency transducers (2–5 MHz), which are usually employed in routine US, high-frequency transducers (5–7 MHz) provide excellent images of the internal structure of GPLs [7], but penetration tends to be shallow. However, recent advances in US technology have allowed high-frequency transducers to provide resolutions of greater than 8 cm, which can cover the entire GB. High-frequency transducers can also delineate the GB wall as a two- or three-layer structure: The inner hypoechoic layer and outer hyperechoic layer or hyperechoic inner and outer layers, and a hypoechoic middle layer.

**Figure 2:**
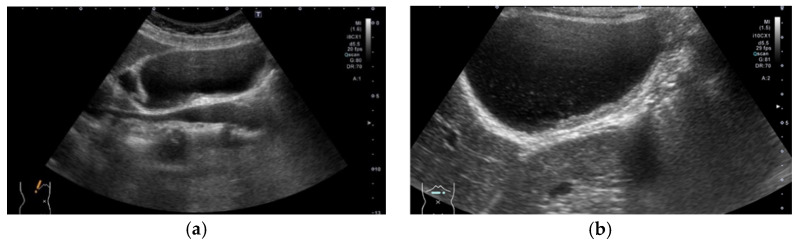
Evaluation of GB using a HRUS technique. Compared with an image of routine US (**a**), localized slight thickening of an inner hypoechoic layer was clearly shown in the magnified image using a high-frequency transducer (**b**) (GBC case).

High-resolution US (HRUS) is a useful technique, in particular for detecting small lesions, which use both low- and high-frequency transducers during evaluation [8]. Kim et al. [8] reported that the sensitivity and specificity for diagnosing malignant polyps using HRUS were 66.6% and 89.2%, respectively. Joo et al. [9] showed that HRUS can be helpful for distinguishing adenomyomatosis (ADM) from early-stage wall-thickening-type GBCs. Therefore, for differential diagnosis, additional detailed evaluation with magnified images using high-frequency convex and linear transducers is essential (Figure 2).

## 3. Morphological Classification of US Appearance

**Figure 3:**
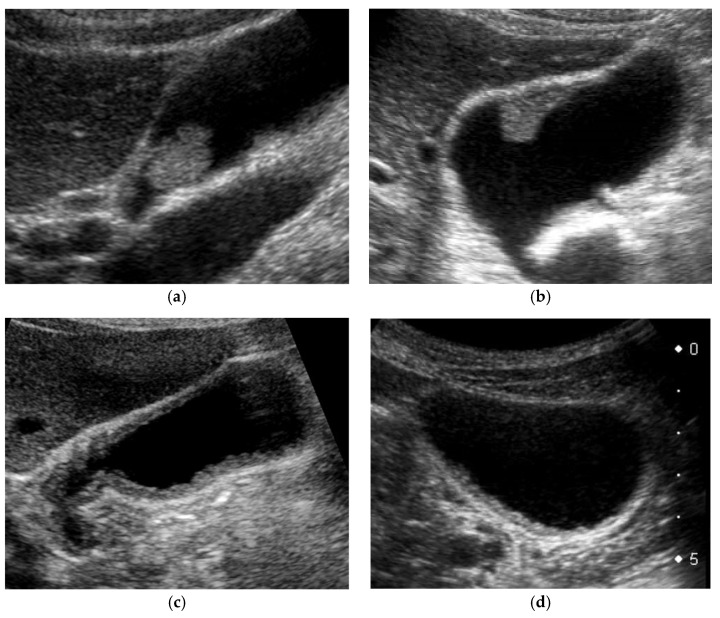
Morphological classification of US appearance. (**a**) Pedunculated GPL, (**b**) sessile (broad-based) GPL, (**c**) diffuse GWT, (**d**) focal GWT (all cases are GBCs).

US appearance of GB lesions is broadly divided into polypoid lesions (GPLs) and wall thickening (GWTs) (Figure 3). This classification is useful for differential diagnosis and is also used in US cancer screening [1].

GPLs are defined as focal elevation or protrusions that are distinguishable from the surrounding mucosa, and can be classified into pedunculated and sessile (broad-based). This classification is useful not only for the differential diagnosis, but also for assessing the depth of invasion. Pedunculated carcinomas are considered to be confined to the mucosal layer (M) [10,11,12]. However, sessile (broad-based) carcinomas include early-stage cancers as well as advanced cancers that invade deeper than the subserosa (SS). The most important targets to be differentiated are cholesterol polyps in the pedunculated type and adenomyomatosis (ADM) in the sessile type. For GPLs that are difficult to classify, it is recommended to check for polyp shape change due to postural changes. In addition, contrast-enhanced US (CEUS) can accurately diagnose pedunculated lesions, which showed a sessile shape on the conventional B-mode (pseudo-sessile-shaped polyp) [13]. Three-dimensional US can also facilitate the morphological recognition of GPLs [14]. If the classification is still difficult, the lesion should be classified as sessile with a high frequency of malignant lesions.

**Figure 4:**
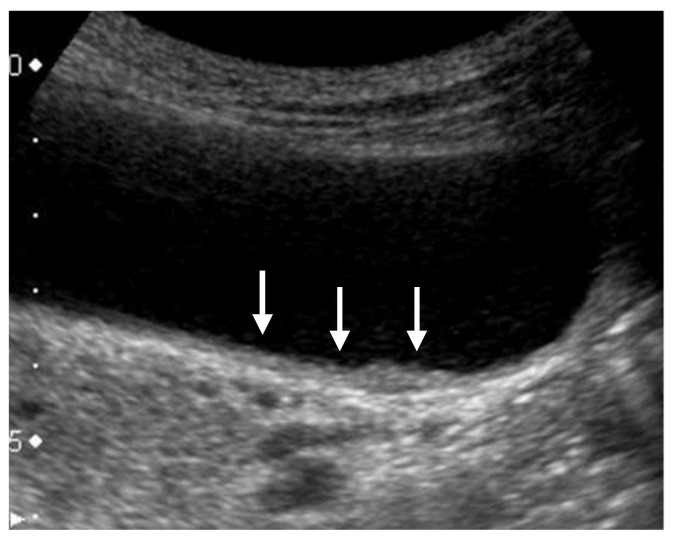
Focal presence of an inner hypoechoic layer (focal GWT). A high-frequency transducer with magnified image showed a slightly thickened inner hypoechoic layer less than 4-mm thick (arrow) in the fundus of GB (GBC case).

GWTs should be determined as wall thickening of 4 mm or more, and subdivided into diffuse and focal [1]. The presence of a partial inner hypoechoic layer of less than 4 mm in thickness (Figure 4) should also be included in focal GWT [1], as it may correspond to early-stage wall-thickening GBC. Diffuse GWT is not a specific US finding for GB abnormalities such as cholecystitis and GBCs; it can also occur in systemic diseases or inflammation of organs adjacent to the GB. However, focal GWT is a specific finding for the GB and is associated with a high frequency of malignant lesions. As GWTs, especially focal lesions, are more difficult to pick up than GPLs, magnification with a high-frequency transducer is strongly recommended (Figure 2). In addition, US evaluation should be performed after appropriate fasting time to rule out the effect of the postprandial contraction.

## 4. Differentiation of GB Polypoid Lesions (GPLs)

Classification into pedunculated and sessile (broad-based) types (Figure 3a,b) is essential for differential diagnosis and assessment of the depth of invasion. Pedunculated lesions include the most common cholesterol polyps, early GBCs, adenomas, inflammatory polyps, and hyperplastic polyps. However, sessile lesions include early GBCs, advanced GBCs, localized ADM, and sludge. Sessile shape is a strong predictive factor of GBCs [15,16,17,18,19] and includes both early-stage and advanced GBCs, whereas GBCs are less frequent in pedunculated GPLs and all cases are confined to the mucosal layer [10,11,12]. According to a systematic review [17], the odds of malignancy in sessile GPLs are increased by a factor of 7.32 (95% confidence interval 4.18–12.82). In GPLs more than 10 mm, sessile shape (OR, 9.485–41.257) was a significant predictor of differentiating GBCs from adenomas (*p* < 0.05) [18].

This classification is useful for differential diagnosis and has been adopted for US cancer screening [1]. However, since most previous reports have evaluated GPLs without separating pedunculated and sessile lesions, this review also discusses the findings of GPL including both types, except for a few findings.

### 4.1. Multiplicity

The distribution of single polyps has been reported to be 50.7–89.5% [3], and several studies have reported that a solitary lesion is a common US finding for neoplastic polyps [8,15,16,17,18,19]. A study using cholecystectomy specimens showed that only 25% of cholesterol polyps were solitary, compared to 88% of adenomas and GBCs [15]. Solitary polyps are associated with a 2.05-fold (95% confidence interval 1.52–2.75) increased probability of malignancy in a systematic review [17]. In addition, in GPLs larger than 10 mm, a single polyp (OR, 3.680–3.856) was also associated with neoplastic polyps (*p* < 0.05) [18].

### 4.2. Size

The size of pedunculated GPLs has been divided into 5 mm or less than 5 mm, 6–10 mm, and more than 10 mm for differential diagnosis in US cancer screening [1], and the distribution of GPLs has been reported to be 50%, 16.4–42.1%, and 0–2.3%, respectively [3]. Several previous studies have reported that the size of GPLs is deemed a predictive US finding for malignant potential [8,15,16,17,18,19,20,21]. This finding was first reported by Kozuka et al. [20], who determined that a final pathological size of 12 mm could separate benign adenomas from adenomas with malignant changes and invasive GBCs. Kubota et al. [15] reported that the mean sizes of cholesterol polyps, adenomas, carcinomas, and inflammatory polyps of cholecystectomy specimens were 8.8, 6.9, 25.7, and 4 mm, respectively. These data suggest that a size of 10 mm can be used for the differential diagnosis. However, even polyps smaller than 10 mm can be malignant [19], and most polyps larger than 10 mm are also benign.

Some current guidelines recommend cholecystectomy for GPLs that are 10 mm or larger than 10 mm. Park et al. [21] calculated the AUC using the ROC curve to validate the conventional size criteria. The sensitivity and specificity for predicting malignant polyps were 98.2% and 19.6% at 10 mm and 91.0% and 71.8% at 13 mm, respectively. Since GPLs in the size range of 10–12 mm were unlikely to be malignant, surgical indication for GPLs larger than 13 mm can prevent 50% of unnecessary cholecystectomies without the risk of missing malignant GPLs. According to a clinicopathological study [22], 90% of polyps 10 mm or larger than 10 mm in size are neoplastic. However, since 30% of neoplastic polyps are smaller than 10 mm, even small polyps need to be carefully monitored, especially in elderly patients.

Regarding the growth rate, the percentage of GPLs that showed growth over the follow-up period ranged from 1% to 23% [19]. Kubota et al. [15] reported that all GPLs with an increase in maximum diameter (1.5–4 times over 4–12 months) were GBCs. Although it is difficult to find a specific growth rate that suggests an increased risk of malignancy, a rapid change in size should raise the suspicion of malignancy. An initial review within 6 months is recommended to assess polyp growth.

### 4.3. Surface Contour

Understanding the pathological features is important in interpreting US images of GPLs. According to a pathological study, a nodular surface contour with relatively shallow notches is characteristic of neoplasms, while granular components with relatively deep notches suggest non-neoplastic GPLs [23]. Kim et al. [8] divided the surface contour of GPLs into smooth and lobulated types using HRUS and found that the smooth surface was significantly common in non-neoplastic polyps and lobulated surface was significantly common in neoplastic polyps. Choi et al. [24] also divided the polyp surface into lobulated and non-lobulated using endoscopic ultrasound (EUS), and reported that lobulated surface structures were more common in neoplastic lesions in both univariate (*p* < 0.01) and multivariate analyses (*p* = 0.03). Based on these reports, nodular or lobulated patterns should be considered neoplastic. However, Sugiyama et al. [25] examined the surface of cholesterol polyps by size and reported that the surface of 10 mm or smaller cholesterol polyps showed smooth (55.9%), granular (35.3%), or nodular patterns (8.8%), while the polyp surface larger than 10 mm was granular (85.7%.), smooth (7.2%), or nodular (7.2%). Thus, the sensitivity of smooth pattern for non-neoplastic GPLs depended on size, and the outline of GPLs within 11–20 mm could not distinguish between malignant and benign GPLs [13].

### 4.4. Internal Structure

#### 4.4.1. Hyperechoic Spots and Aggregation of Echogenic Spots (Mulberry Echo Pattern)

**Figure 5:**
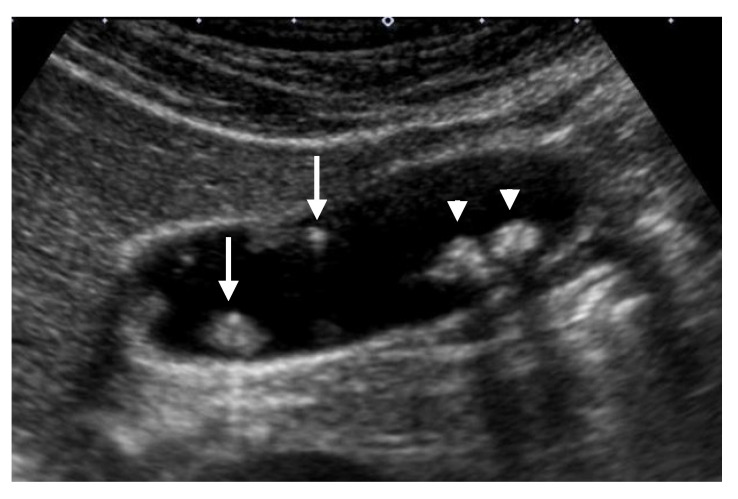
Hyperechoic spots and aggregations of echogenic spots (mulberry echo pattern). A high-frequency transducer depicted hyperechoic spots (arrow) and aggregations of echogenic spots with acoustic shadow (arrowhead) (cholesterol polyps).

Most cholesterol polyps are accompanied by a tiny hyperechoic spot or aggregation of echogenic spots (mulberry echo pattern) (Figure 5) [8,18,24,25,26,27], which represent a mass of foamy histiocytes containing cholesterol. As both findings showed high specificity (100%) for cholesterol polyps [26], these findings are considered benign US findings in polyps of 6–10 mm in size in US cancer screening [1]. However, US only detected these findings in 60–77.1% of cholesterol polyps [25,26]. Since a tiny hyperechoic spot was more common in 10 mm or smaller cholesterol [25], the presence of a hyperechoic spot could not distinguish between malignant and benign GPLs within 11 to 20 mm [13]. Furthermore, there is some possibility that neoplastic polyps associated with cholesterolosis were more common than we expected [24,28]. Thus, hyperechoic spots alone may not be predictive of cholesterol polyps. Sadamoto et al. [27] proposed a scoring system formula using the maximum diameter, internal echo patterns and hyperechoic spots. The sensitivity and specificity at a cut-off score of more than 12 were 77.8% and 82.7%, respectively.

#### 4.4.2. Cystic Structures (Anechoic Spots), Comet Tail Artifacts, Echogenic Foci

Cystic structures (anechoic spots) reflect two distinct pathological abnormalities: Dilated atypical (neoplastic) glands, especially in well-differentiated GBCs [7,29,30,31], and Rokitansky–Aschoff sinuses (RAS) in ADMs. High-frequency transducers, especially linear probes with a magnified image, can more precisely delineate these internal structures [7].

**Figure 6:**
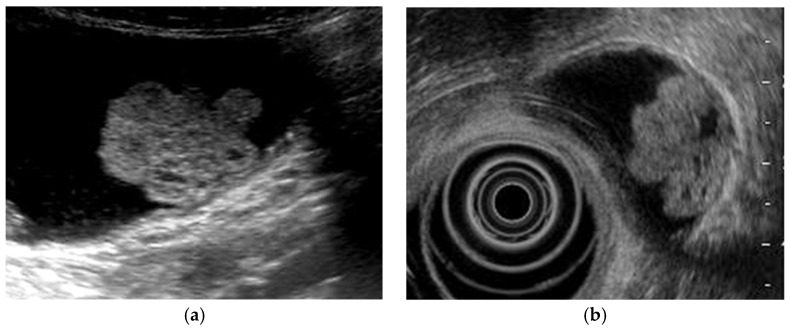
Cystic structures (anechoic spots) corresponding to cystically dilated cancerous glands. (**a**) High-frequency transducer (8 MHz) and (**b**) EUS (12 MHz) showed irregularly shaped cystic lesions of various sizes corresponding to cystically dilated cancerous glands (GBC concomitant with pancreatobiliary maljunction case).

Niizawa et al. [29] were the first to report small anechoic spots in adenomas corresponding to dilated ductal structures. Noda et al. [30] also reported that cystic lesions about 2 mm in size were observed in 63.6% of adenomas, which histologically corresponded to cystically dilated atypical glands. These lesions, unlike RAS, appear as irregularly shaped and unequal sized cystic lesions (Figure 6). Yoshimitsu et al. [31] reported well-differentiated GBCs with intratumoral cystic components due to abundant mucin production using MRI. They suggested that RAS in ADM was larger in number, round in shape, and aligned in a linear fashion, whereas cystic components of GBCs were multilobulated in shape, larger in size, and had an irregular surface.

In ADM, multiple microcystic components correspond to proliferated RAS [32], which is considered a highly specific finding [26,33]. However, the sonographic appearance of cystic lesions varies depending on their size, location, and contents, and the diagnostic accuracy of conventional US has been reported as less than 70% [34]. In addition to cystic structures, comet-tail artifacts and echogenic foci, which also represent minute cystic lesions and intramural calculus inside RAS are also useful [26]. Furthermore, twinkling artifacts on Doppler image facilitate the detection of RAS containing tiny echogenic foci that commonly cause a reverberation artifact.

**Figure 7:**
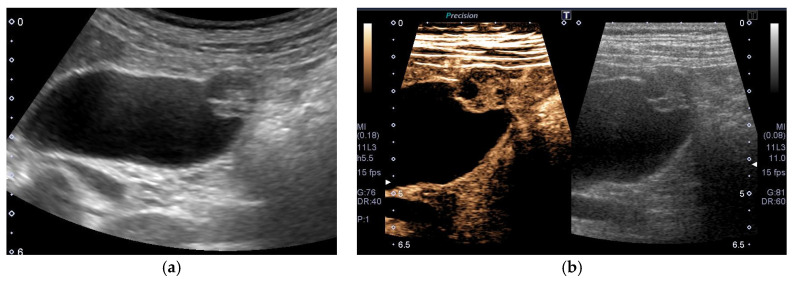
Cystic structures (anechoic spots) corresponding to RAS. Compared to the conventional US (**a**), CEUS clearly depicted cystic structures inside the fundal ADM (**b**).

CEUS can detect RAS better than conventional US, because RAS remains anechoic while the GB wall is enhanced (Figure 7). Tang et al. [35] reported that a small non-enhancement space observed in both arterial and venous phases was a characteristic finding of ADM, and CEUS (100%) could increase the degree of visualization of RAS compared with conventional US (67%). Yuan et al. [36] reported that intramural anechoic space was detected in 56.1% of focal ADM in contrast to 20.6% of GBC (*p* = 0.002).

**Figure 8:**
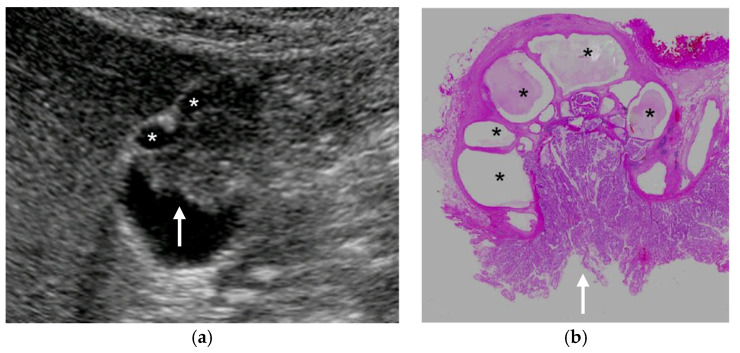
GBC concomitant with localized ADM. (**a**) US showed a papillary GPL (arrow) with dilated cystic lesions in the fundus. (**b**) Histopathologically, a papillary GBC (arrow) arose in the surface mucosa of localized ADM and cystic lesions were corresponding to dilated RASs (*: dilated RAS). Reprinted with permission from ref. [7]. Copyright 2021 Japan Society of Ultrasonics in Medicine.

Recently, several GBCs concomitant with fundal type ADM have been reported [7,35,37,38,39,40,41] (Figure 8). These GBCs showed well-circumscribed papillary growth just above the surface mucosa of ADM or inside RAS. Therefore, even in cases with the presence of RAS, the mucosal surface of ADM and inside RAS should be evaluated in detail to detect concomitant GBCs. Tang et al. [35] reported that the discontinuity of the wall on CEUS was helpful for the diagnosis of ADM with GBC.

### 4.5. Stalk Width

In pedunculated GPLs, the stalk width is another useful finding. Cholesterol polyps usually present with a “ball on the wall sign” [42] because their stalks are thin and cannot be detected by conventional US. The “flickering sign”, which is due to the pulsation of vena cava is also a characteristic finding of pedunculated GPLs with a thin stalk and suggestive of cholesterol polyps [7]. Recently, a highly sensitive Doppler imaging modality (superb microvascular imaging (SMI) in the Aplio series (Canon Medical Systems, Otawara, Japan) and B-flow (GE Healthcare, Ltd., Chicago, IL, USA)) has been developed and can depict a minute vessel in the stalk of a benign polyp without contrast agent administration [43]. On CEUS, adenomas (0.56 ± 0.48 mm) demonstrated a significantly wider stalk width compared to cholesterol polyps (0.23 ± 0.59 mm), and the stalk width of GBCs was greater than that of benign polyps [44].

### 4.6. Localized Slight Thickening of Inner Hypoechoic Layer around GPLs

**Figure 9:**
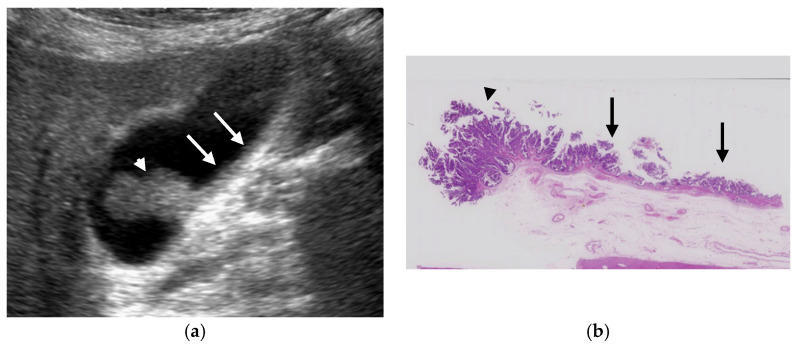
Localized slight thickening of an inner hypoechoic layer around GPL. (**a**) HRUS delineated a localized slight thickening of an inner hypoechoic layer (arrow) around a sessile GBC (arrowhead). (**b**) Histopathologically, this finding corresponded to the laterally spreading of carcinoma (arrow). Reprinted with permission from ref. [7]. Copyright 2021 Japan Society of Ultrasonics in Medicine.

The progression of GBCs includes lateral spreading on the mucosal surface (horizontal progress) and deep invasion in the wall (vertical invasion). Lateral spreading is the main route in early-stage GBCs. According to a clinicopathological study on early GBCs, 10% of pedunculated and 75% of sessile GBCs were accompanied by laterally spreading components [10]. Eguchi et al. [45] reported that 66% of early GBCs had superficial spread of carcinoma, and sessile type or superficial raised type was more associated with superficial spreading than pedunculated type. Wakai et al. [46] also reported that 88% of sessile early-stage GBCs were accompanied by superficially elevated and/or flat tumors. US, especially with a high-frequency transducer with zoom magnification can detect localized slight thickening of the inner hypoechoic layer around GPLs (Figure 9), corresponding to the lateral spreading of flat-type GBCs [7,47].

### 4.7. Irregularity or Discontinuity of GB Wall Layer Structure

High-frequency transducers can delineate GB wall as two- or three-layer structure: An innermost hypoechoic layer and an outermost hyperechoic layer or an innermost hyperechoic layer, a middle hypoechoic layer, and an outermost hyperechoic layer. According to a precise comparison of the layer structure of EUS and its histology [48,49], the inner hypoechoic layer includes not only the mucosa and muscularis propria but also the fibrous layer of the subserosa. Therefore, the irregularity of the outer hyperechoic layer of the adjacent wall suggests GBC with invasion into the adipose layer of the subserosa (T2), and the disruption of the outer hyperechoic layer suggests GBC with invasion beyond subserosa [11]. However, sessile GPLs with an intact outer hyperechoic layer include not only non-neoplastic GPLs but GBCs confining to the mucosa (T1a) or muscularis propria (T1b), or have invaded the fibrous layer of the subserosa (T2).

In most cases, the characteristics of the GB wall can be evaluated at the boundary between the liver and GB wall using conventional US. CEUS is highly useful for observing the continuity of the GB wall, especially in cases with inflammation or biliary sludge and improve the diagnostic performance. The discontinuity of the GB wall on CEUS showed the high sensitivity and specificity (82% and 93%, respectively) [50]. Xie et al. [51] demonstrated that the disruption of the GB wall on CEUS was the best indicator of malignant lesions with the highest sensitivity and specificity (85% and 100%, respectively).

### 4.8. Blood Flow Analysis and Contrast Effect

**Figure 10:**
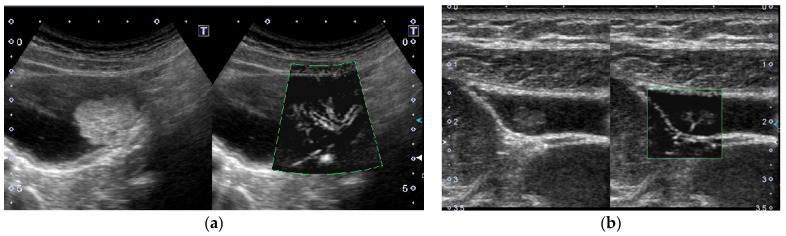
Vascular imaging using superb microvascular imaging (SMI). (**a**) Multiple flow signals spreading dendritically from the base into the lesion were delineated in a GBC. (**b**) A branched signal was detected in a cholesterol polyp.

Color Doppler imaging is used to detect vessels inside GPLs and GB wall, and evaluate its direction. Since the most recent highly-sensitive Doppler imaging can detect low-flow signals, minute vessels in GPLs can be evaluated without using contrast agents (Figure 10). In previous reports, color signal patterns have been roughly classified into absent, diffuse, dotted, linear, irregular, branched, tortuous, arborizing, and so on. Hirooka et al. [52] reported that color signal patterns of GBCs were diffuse or arborizing (sensitivity 90.5% and specificity 62.5%).

As for CEUS, Miwa et al. [13] proposed shape (regular or irregular) and thickness (dilated or thin) as parameters for classifying vessels: Vessels of GBC were irregular in 82% and dilated in 71%, whereas in cases of benign polyps, 95% had regular and 89% had thin vessels. Dilated vessels, characterized by caliber changes of approximately 1 mm in diameter on CEUS, were significantly correlated with malignant GPLs (*p* < 0.001) [13]. Kin et al. [53] reported that contrast-enhanced SMI provided a microvascular image with good quality compared with SMI, and tortuous microvessels and the presence of abrupt caliber change in microvessels showed a significant difference between benign and malignant GB lesions (*p* = 0.032, *p* < 0.001, respectively). However, irregularly shaped signal patterns and dilated blood vessels were detected even in non-neoplastic GPLs in a few cases [13,52,53]. Measurement of GB wall blood flow (GWBF) is another clue for differentiation. GBC showed a significantly rapid blood flow value compared with other GB abnormalities and healthy volunteers. When the cut-off level of GWBF velocity was set at 30 cm/s, GBC could be diagnosed with 100% sensitivity and 96% specificity [54].

On CEUS, several reports have evaluated the perfusion effect in the arterial phase (early phase) and venous phase (late phase). However, since GBC, adenoma, inflammatory polyp, and even cholesterol polyp show mild or marked tumor enhancement in the arterial phase, it is considered difficult to differentiate GPLs except biliary sludge using the perfusion effect in the arterial phase. On the other hand, in the venous phase of CEUS, GBC has been reported to show hypoenhancement compared to benign polyps [50,51,55]. This “rapid wash-out change” might be related to the abundant blood supply in malignant lesions, though there are fewer histological explanations. However, the timing for assessing the degree of enhancement in the venous phase was not the same among previous reports. Therefore, it is controversial to evaluate the usefulness of the “rapid wash-out change” in the differential diagnosis of GPLs.

Heterogeneous enhancement is generally used for the differential diagnosis of liver tumors, and also useful for characterizing GPL [13,55]. Since most hypervascular lesions usually show homogeneous enhancement in the arterial phase, the heterogeneity of enhancement should be evaluated in the venous phase. GBC shows a heterogeneous contrast effect by variate vessel shape and diameter, whereas adenoma and benign polyps show homogeneous enhancement [56].

For the differential diagnosis of GPLs, the classification into pedunculated and sessile is essential. Internal structures such as hyperechoic spots, aggregation of echogenic spots, and cystic structure are highly specific for cholesterol polyps and ADM, respectively. After assessing these findings using a high-resolution transducer, it is recommended to assess the GWBF and shape and caliber change of color signal patterns on Doppler imaging. If CEUS is available, it is also useful to evaluate enhanced pattern in the venous phase (late phase) (Table 1).

## 5. Differentiation of GB Wall Thickenings (GWTs)

The normal GB wall is composed of four layers: Mucosa, muscularis propria, subserosa, and serosa. Under favorable conditions, US, especially when employing high-frequency transducers, can identify two- or three-layers: An innermost hypoechoic layer and an outermost hyperechoic layer or an innermost hyperechoic layer, a middle hypoechoic layer, and an outermost hyperechoic layer. According to the precise comparison of the US layer of the GB wall and histological structure [48,49], the inner hypoechoic layer in a two-layer structure and the hypoechoic middle layer in a three-layer structure include not only the mucosa and muscularis propria but the fibrous layer of the subserosa. The outermost hyperechoic layer represents the serosa and the adipose layer of the subserosal.

GWTs include wall thickening of 4 mm or greater and focal presence of a partial inner hypoechoic layer of the GB wall even if the thickness is less than 4 mm [1] (Figure 3). Kim et al. [57] classified GWT of xanthogranulomatous cholecystitis (XGC) into diffuse (>50%) and localized (<50%) according to the extent of thickening. Thus, diffuse GWT was defined as a condition in which more than 50% of the GB wall shows wall thickening of 4 mm or more (Figure 3c), whereas focal GWT includes not only the wall thickening of 4 mm or more in less than 50% of the GB wall (Figure 3d), but also the focal presence of the inner hypoechoic layer, even if the wall thickness is less than 4 mm.

Diffuse GWTs can be seen in the extracholecystic inflammation, hepatic disorders, and systemic diseases, as well as GB disease, and pseudo-thickening is included (Table 2), whereas focal GWTs are specific for GB disorders and the incidence of GBCs is higher. Lee et al. [58] reported that non-focal wall thickening is a statistically common finding for XGC compared to GBC (*p* < 0.001). Cui et al. [59] reported that focal thickening of the GB wall was more frequent in XGC patients concomitant with GBC than those without GBC (*p* = 0.0117).

This classification is highly useful for differential diagnosis. However, since most previous reports have examined GWTs without separating diffuse and focal types, this review also discusses findings of GWTs including both types, except for a few findings.

### 5.1. Layer Structure

#### 5.1.1. Characteristics of Innermost Hyperechoic Layer (IHL)

The source of the innermost hyperechoic layer (IHL) is considered mostly interface echoes [49], and the mucosa is also included [9]. In the acute stage of cholecystitis, thickening of the GB wall is associated with congestion and edema. Therefore, the mucosal surface is well defined and “three-layer” thickening is often present. Thickening of the GB wall unrelated to GB condition, including congestive heart failure, renal failure, liver disease (hepatic failure, hepatitis), ascites, hypoalbuminemia, and pancreatitis, was also due to edema [60]. However, GBCs arise from the GB epithelium and cause mucosal irregularity and disruption in most cases. These differences in the mucosal surface affect the characteristics of IHL.

The discontinuity of the mucosal echoes, which includes focal or diffuse loss of the normal specular echo from the GB mucosa occurred in 62% of patients with GBC, compared with only 10% of patients with benign GB conditions (*p* = 0.05) [61]. According to a comparative study between ADM and early-stage, wall-thickening-type GBC using HRUS, focal IHL discontinuity and IHL irregularity were found in 4.4% and 11.1% of patients with ADM and 39.3% and 85.7% of patients with GBC and were significantly different (*p* < 0.001) [9]. Furthermore, Lee et al. [58] reported that the continuity of the mucosa was a statistically common finding for XGC compared to GBCs (*p* < 0.05). Multiple logistic regression analysis revealed that the inner layer discontinuity on CEUS was an independent predictor of malignant GWT and achieved the highest diagnostic performance in ROC analysis [62].

**Figure 11:**
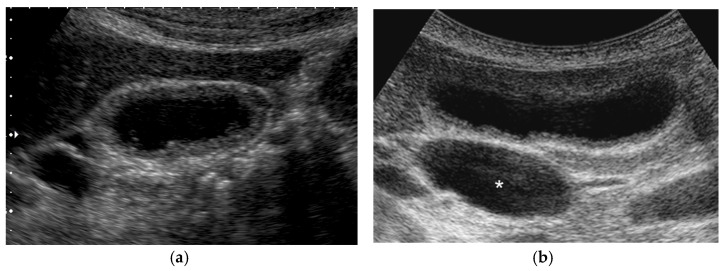
Diffuse GWT without IHL in pancreaticobiliary maljunction (PBM). US showed thickening of the inner hypoechoic layer without the presence of IHL and outermost hyperechoic layer. (**a**) PBM without biliary dilatation, (**b**) PBM concomitant with biliary dilatation (*: dilated extrahepatic bile duct).

Pancreaticobiliary maljunction (PBM) is a congenital anomaly defined as a junction of the pancreatic and bile ducts located outside the duodenal wall and a risk factor for GBC development via the hyperplasia–dysplasia–carcinoma sequence. The incidence of mucosal hyperplasia of PBM patients without biliary dilatation was reported to be 72% [63] to 91% [64]. The GB wall appeared as thickening of the inner hypoechoic layer without IHL and outermost hyperechoic layer (Figure 11). Histologically, the GB of PBM without biliary dilatation showed wall thickness composed of epithelial hyperplasia (88%), hypertrophic muscular layer (63%), and subserosal fibrosis (88%) [65].

**Figure 12:**
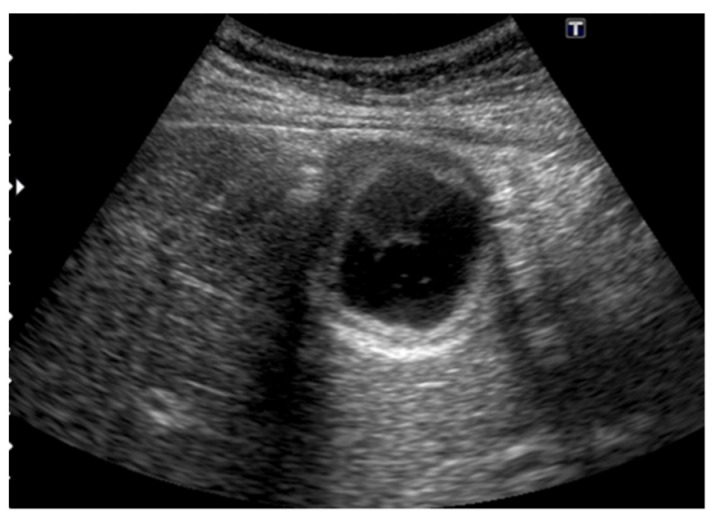
Thickening of IHL. US showed thickened IHL and irregular layer structures of diffuse GWT (GBC case).

The thickening of IHL is also useful for differentiation. When low papillary tumors aggregate on the mucosal surface, distortion of mucosal structures and echo scattering may occur, indicating thickening of IHL (Figure 12). According to a study using HRUS, IHL thickening greater than 1 mm was significantly associated with GBCs (*p* < 0.05) [9].

#### 5.1.2. Sonolucent Layer, Hypoechoic Zone, “Three-Layer” Thickening

Wall thickening in acute cholecystitis presents relatively uniform thickness of layer structure, showing as a sonolucent layer between two echogenic lines in the GB wall. A hyporeflective or sonolucent layer within the thickened GB wall probably reflects the subserosal edema and necrosis [66]. Submucosal echolucency was significantly more common in patients with benign GB disease than in patients with GBC (*p* = 0.01) [61]. However, a wall sonolucency was seen in only 39% of acute cholecystitis and 4% in chronic cases [67]. A “three-layer” thickening, which consists of a single circumferential lucent zone between two relatively uniform echogenic layers is also used as the same finding. Cohan et al. [68] reported that the sensitivity and specificity of a “three-layer” thickening for acute cholecystitis were only 8% and 71%, respectively.

#### 5.1.3. Striations

Striations are striated wall thickening, consisting of several alternating, irregular, discontinuous, lucent, and echogenic bands, and observed diffusely or focally in the GB wall. Striations, which are due to edema of the GB wall, have been considered strong evidence for acute cholecystitis, showing the sensitivity of only 62%, but the specificity of 100% [68]. Unlike uncomplicated acute cholecystitis, in which inflammation is limited primarily to the submucosal layer of the GB wall, coagulative necrosis often involves all layers of the GB wall in XGC. Therefore, the presence of striations in acute cholecystitis was suggestive of gangrenous changes in the GB wall [60].

However, striations may occur in various diseases, including congestive heart failure, renal failure, liver disease (hepatic failure, hepatitis), ascites, hypoalbuminemia, and pancreatitis [60,69]. Hepatitis A virus infection (odds ratio = 3.17 [1.42–7.09]), female gender (odds ratio = 2.47 [1.34–4.56]), and an elevated total bilirubin level (odds ratio = 1.09 [1.03–1.15]) were positively associated with GWT secondary to hepatitis [69]. The enlarged GB cavity is characteristic of cholecystitis and is useful in differentiating striations due to other causes.

#### 5.1.4. Irregularity or Discontinuity of Layer Structure, Irregular Thickening of Outer Hyperechoic Layer

The wall thickening due to edema, including acute cholecystitis and systemic diseases, shows a relatively uniform thickness and appears as a “three-layer” layer structure: An innermost hyperechoic layer, a middle hypoechoic layer, and an outermost hyperechoic layer. However, XGC is difficult to differentiate from GBC because the interface with the liver is often lost due to the characteristic pathological pattern of lipid-containing histiocytes infiltrating

**Figure 13:**
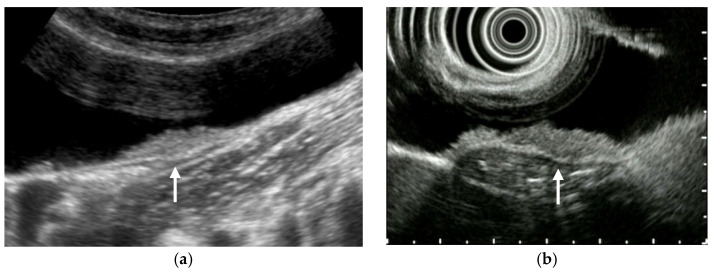
Discontinuity of an outer hyperechoic layer. A high-frequency transducer showed the discontinuity of an outer hyperechoic layer (arrow) in focal GWT (**a**), EUS also delineated the thinning of an outer hyperechoic layer (arrow) (**b**) (GBC case).

Typical US findings in wall thickening GBC include hypoechoic, irregular wall thickening due to tumor invasion. According to a study using HRUS, loss of the multiple layer pattern was detected in 64.3% of wall-thickening-type GBC [9]. Furthermore, tumor invasion into the adipose layer of the subserosa causes the irregularity of the outer hyperechoic layer and tumor invasion beyond the subserosa causes the discontinuity of the outer hyperechoic layer [49] (Figure 13). On CEUS, the destruction of the GB wall intactness was absent in benign diseases, and the sensitivity, specificity, and accuracy in the differential diagnosis between malignant and benign GB diseases were 84.8%, 100%, and 93.8%, respectively [51]. According to a systematic review of CEUS, discontinuity of the GB wall in differentiating benign and malignant diseases showed the highest sensitivity (82%) and specificity (93%) among all malignant features on CEUS [50].

An irregularly thickened outer hyperechoic layer is also useful for differential diagnosis. Joe et al. [9] reported that the sensitivity and specificity of irregular thickness of an outer hyperechoic wall for the diagnosis of ADM or wall-thickening-type GBC were 57.1% and 88.9%, respectively.

### 5.2. Internal Structure

#### 5.2.1. Cystic Structures (Anechoic Spots), Comet-Tail Artifacts, Echogenic Foci

Cystic structures (anechoic spots) and comet-tail artifacts reflect RAS and characteristic findings of ADM. However, conventional US had an accuracy of only 66% for the diagnosis of ADM [34]. However, HRUS has been reported to delineate cystic lesions with a sensitivity of 51.1% even in small cystic lesions of less than 3 mm in diameter [9]. The sensitivity, specificity, and accuracy of intramural cystic spaces/echogenic foci for the diagnosis of ADM using HRUS were 80.0%, 85.7%, and 82.2%, respectively [9]. On CEUS, small anechoic spaces observed in both arterial and venous phases were characteristic finding of ADM. Tang et al. [35] demonstrated that the sensitivity of CEUS (100%) in the detection of RAS in focal type of ADM was significantly higher than conventional US (66.7%).

**Figure 14:**
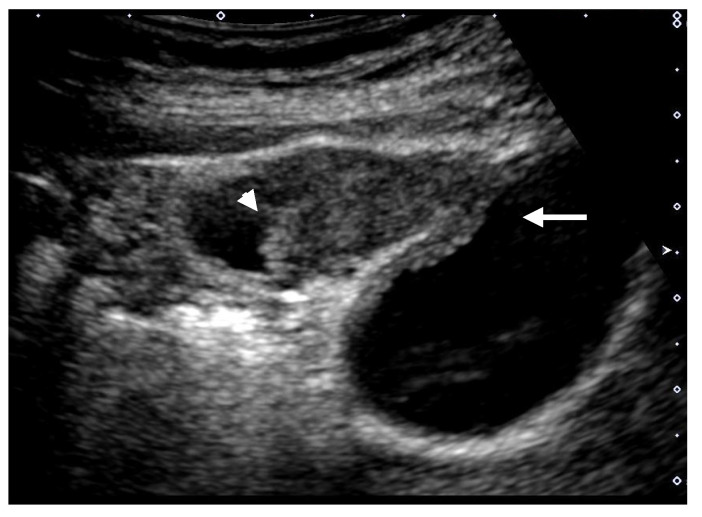
GBC concomitant with segmental ADM. A high-frequency transducer delineated a hypoechoic sessile GBC (arrowhead) in the fundus distal to the annular stricture (arrow).

Segmental ADM is a high-risk condition for GBC [41,70,71] (Figure 14), especially in patients older than 60 years old [70,71]. Since epithelial metaplasia was more marked in the fundal mucosa of segmental ADM than in the neck mucosa (*p* = 0.003) [70], GBC arose in the mucosa of the fundal compartment distal to the annular stricture of the segmental type ADM [71].

#### 5.2.2. Hypoechoic Nodules and Bands

XGC is characterized by distinct pathological findings such as fat-laden macrophages and foamy histiocytes and is associated with severe fibrosis. Several reports [57,58,72] suggested that hypoechoic nodules and bands in the GB wall were the most characteristic findings in XGC. Intramural nodules were discrete, oval or flat, with low echogenicity, and ranged in size from 6 to 12 mm (mean, 10.5 mm) [57]. Lee et al. [58] reported that intramural nodules were found in 70.8% of XGC and 31.6% of GBCs on HRUS, and a statistically more common finding in XGC (*p* = 0.015). A comparative study between US images and pathological findings of the resected specimen showed well-defined hypoechoic areas corresponding to the xanthogranulomatous foci [72]. These lesions were thought to be due to the rupture of the RAS with intramural extravasation of bile and subsequent xanthogranulomatous [73].

### 5.3. Shape of the GB (Symmetrical or Asymmetrical)

In diffuse GWT, diffuse-type ADM involves the whole GB wall symmetrically and segmental ADM narrows the lumen symmetrically. XGC can cause asymmetrical thickening of the GB wall and demonstrate a tendency to form nodules. In focal GWT, most benign lesions were symmetrical (68.8%), while asymmetrical thickening was more common in GBCs (75%, *p* < 0.05) [74].

### 5.4. Cholecystolithiasis

Gallstones are common in acute or chronic cholecystitis and XGC, and are relatively common in GBC and ADM. Ninety-five percent of acute cholecystitis is due to an obstructing calculus in the neck of the GB or cystic duct, while stones are usually absent in diffuse GWTs secondary to acute hepatitis, liver cirrhosis, heart failure, renal failure, and hypoalbuminemia.

Wibbenmeyer et al. [61] reported that cholelithiasis was present in 85% of unsuspected GBCs, and a large solitary gallstone (*p* = 0.005) and a displaced stone (*p* = 0.01) were statistically significantly more common in patients with unsuspected GBC. According to the study of cholecystectomy cases, the prevalence of cholecystolithiasis was higher in patients with segmental ADM (88.9%) than in those without ADM (52.3%; *p* < 0.001), especially in cases with hourglass deformity [75]. Lee et al. [58] compared the diagnostic performance of HRUS, CT, and MRI in differentiating between XGC and GBC, and reported that co-existence of gallstones (OR = 16.5) was independently associated with XGC (*p* = 0.013).

### 5.5. Blood Flow Analysis

Color Doppler is useful in the evaluation of inflammatory disorders including vasodilatation and increased flow. Patients with acute cholecystitis had an abnormally increased flow in the distal two-thirds of the thickened GB wall; however, patients with chronic cholecystitis and necrotizing cholecystitis did not show increased flow [76]. Joo et al. [9] reported that intralesional vascularity on color Doppler imaging was significantly associated with wall-thickening-type GBC and was a negative predictor of ADM (*p* < 0.001).

**Figure 15:**
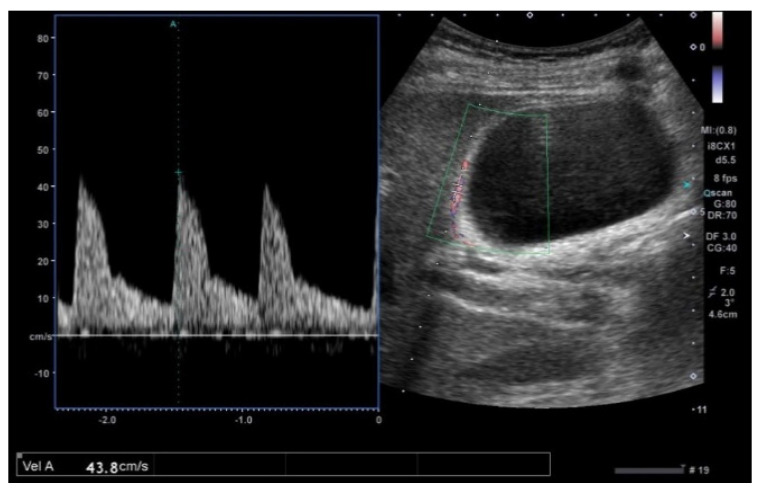
GWBF velocity. GWBF velocity showed 43.8 cm/s in acute cholecystitis.

GWBF velocity in acute cholecystitis (28.6 ± 7.1 cm/s) showed a tendency to be faster than chronic cholecystitis (20.8 ± 4.6 cm/s) and ADM (16.8 ± 4.8 cm/s) [54] (Figure 15). GBC (49.4 ± 12.6 cm/s) showed significantly higher GWBF velocity (*p* < 0.01), and the sensitivity and specificity with the cut-off level at 30 cm/s were 100% and 96%, respectively [54]. Kawashima et al. [77] reported that the mean GWBF velocity of PBM (29.4 ± 3.9 cm/s) was significantly higher (*p* < 0.0001; 95% CI 5.48−13.2) than those without PBM (20.1 ± 5.9 cm/s), and the cut-off level appropriate for diagnosing PBM was estimated to be 25 cm/s. They also speculated that cell proliferation enhancing in the GB wall may contribute to a higher GWBF velocity in cases without GBC.

For the differential diagnosis of GWT, the classification into diffuse and focal is essential. For diffuse GWT, evaluation of other organs in addition to the gallbladder is necessary. The discontinuity and irregularity of the innermost hyperechoic layer and irregular or disrupted GB wall layer structure using a high-resolution transducer suggest GBC. Rapid GWBF is useful not only for the diagnosis of wall-thickened type GBC, but the detection of pancreaticobiliary maljunction (Table 3).

## 6. Conclusions

For the differential diagnosis of GB lesions, morphological classification of US appearance is essential. The diagnostic performance of US can be further improved by combining detailed B-mode evaluation using high-frequency transducers with highly sensitive Doppler imaging and CEUS.

## Figures and Tables

**Table 1:** US findings in major GPLs.

US Findings	cholesterol Polyp	ADM	Adenoma	Carcinoma
US appearance	pedunculated	sessile	pedunculated > sessile	sessile > pedunculated
Multiplicity	multiple > solitary	solitary	solitary	solitary
Surface contour	smooth or granular	smooth or granular	nodular or lobulated	nodular or lobulated
Internal structure	hyperechoic spots, aggregation of echogenic spots	cystic structures (round and smooth surface)	cystic structures (multilobulated and irregular surface)	cystic structures (multilobulated and irregular surface)
Localized slight thickening of inner hypoechoic layer	absent	absent	absent	occasionally
GB wall layer structure	intact outer hyperechoic layer	intact outer hyperechoic layer	intact outer hyperechoic layer	irregular or disrupted outer hyperechoic layer in advanced lesions
Shape of color signal pattern	absent or regular	absent or regular	irregular	irregular
Caliber change of color signal pattern	absent	absent	present	present
GB wall blood flow (GWBF)	lower than 30 cm/s	lower than 30 cm/s	not available	higher than 30 cm/s
Enhanced pattern in the venous phase (late phase)	homogeneous	homogeneous	homogeneous	heterogeneous

**Table 2:** Causes of GB wall thickening (GWT).

		Diffuse GWT	Focal GWT
Gallbladder	Inflammation	Acute cholecystitis	
		Chronic cholecystitis	Chronic cholecystitis
		Xanthogranulomatous cholecystitis	Xanthogranulomatous cholecystitis
	Hyperplasia	Adenomyomatosis (Diffuse, Segmental)	Adenomyomatosis (Focal)
		Hyperplasia associated with pancreaticobiliary maljuncton	
	Neoplasia	Gallbladder carcinoma	Gallbladder carcinoma
		Lymphoma	
	Pseudothickening	Postprandial state	Debris, Sludge
Other organs	Inflammation	Pancreatitis	
		Peritonitis	
	Liver disorders	Acute hepatitis	
		Cirrhosis	
	Systemic diseases	Heart failure	
		Renal failure	
		Hypoalbuminemia	
		Sepsis	

**Table 3:** US findings in major GWT.

US Findings	Acute Cholecystitis	XGC	ADM	Carcinoma
US appearance	diffuse	focal or diffuse	focal or diffuse	focal > diffuse
Innermost Hyperechoic Layer (IHL)	recognized continuously	recognized continuously	recognized continuously	presence of focal or diffuse discontinuity or irregularity
Layer structure	preserved, sonolucent layer, striations	irregular or disrupted in some cases	preserved	irregular or disrupted in advanced lesions
Internal structure	No distinctive findings	hypoechoic nodules and bands	cystic structures (round and smooth surface, aligned in a linear fashion)	cystic structures (multilobulated and irregular surface)
Cholecystolithiasis	common	common	relatively common	relatively common
GB wall blood flow (GWBF)	lower than 30 cm/s (affected by disease activity)	not available	lower than 30 cm/s	higher than 30 cm/s

## Abbreviations

ADM	adenomyomatosis
CEUS	contrast-enhanced ultrasound
EUS	endoscopic ultrasound
GB	gallbladder
GBC	gallbladder carcinoma
GPL	gallbladder polypoid lesion
GWBF	gallbladder wall blood flow
GWT	gallbladder wall thickening
HRUS	high-resolution ultrasound
IHL	innermost hyperechoic layer
PBM	pancreaticobiliary maljunction
RAS	Rokitansky–Aschoff sinuses
SMI	superb microvascular imaging
US	ultrasound
XGC	xanthogranulomatous cholecystitis
